# Deconstructing Self-Compassion: How the Continued Use of the Total Score of the Self-Compassion Scale Hinders Studying a Protective Construct Within the Context of Psychopathology and Stress

**DOI:** 10.1007/s12671-022-01898-4

**Published:** 2022-05-12

**Authors:** Peter Muris, Henry Otgaar

**Affiliations:** 1 Department of Clinical Psychological Science, Faculty of Psychology and Neuroscience, Maastricht University, P.O. Box 616, 6200 MD Maastricht, The Netherlands; 2 Stellenbosch University, Stellenbosch, South Africa; 3 Catholic University Leuven, Leuven, Belgium

**Keywords:** Self-Compassion Scale; Compassionate and uncompassionate self-responding; Psychopathology and stress

## Abstract

In a new commentary in *Mindfulness*, Neff once again tried to defend the use of the Self-Compassion Scale (SCS) total score by arguing that compassionate and uncompassionate self-responding (CS and UCS) are part of a bipolar continuum. In this brief reaction, we refute this notion and also clarify how the continued use of the SCS total score muddies the water of research on self-compassion as a protective variable. We also illustrate how the SCS—by separating CS and UCS—can provide more valid and valuable information on protection and vulnerability within the context of psychopathology and stress than just relying on the total score of the SCS.

In the past years, a heated debate has been going on regarding the validity of the Self-Compassion Scale (SCS; Neff, 2003b) as an index for measuring individual differences in self-compassion, a protective psychological factor that would preserve people’s mental and physical health. On the one side, the scale’s developer, Kristin Neff, maintains the position that the compassionate (CS, i.e., self-kindness, common humanity, and mindfulness) and (reversely scored) uncompassionate self-responding (UCS, i.e., self-judgment, isolation, and over-identification) components included in the scale should be merged into a total score that would yield a reliable and valid index of self-compassion (Neff, 2016a, 2016b, 2019). On the other side, several scholars have repeatedly questioned this procedure and confirmed this with data by pointing out that the inclusion of the UCS components in the SCS is problematic because they represent vulnerability rather than protection, and hence will obscure the pure and unique protective potential of the self-compassion construct (e.g., Brenner et al., 2017, 2018; López et al., 2015, 2018; Muris, 2016; Muris & Petrocchi, 2017; Muris et al., 2016, 2019b).

Muris and Otgaar (2020) summarized the main points of critique with the intention to prompt critical thinking in researchers who are interested in this construct and to terminate the dispute for the time being. Neff (2020) responded to this article by reiterating her arguments and trying to disconfirm our position with flawed arguments, which certainly called for a new reaction. However, we stuck with our decision to end the debate as all main points had already been made. Most importantly, we did agree on one aspect of Neff’s commentary which was the chosen title: “Let the empirical evidence speak on the Self-Compassion Scale” (p. 1900).

Since that time, we have carefully followed the empirical evidence on the Self-Compassion Scale. A notable observation is that the research on self-compassion has continued to flourish. We already noted an exponential increase in publications and citations between 2003, when the construct first appeared in the scientific literature (Neff, 2003a), and 2019 (Muris & Otgaar, 2020), and in the past two years 2020 and 2021, this growth has continued (although the increase in 2021 seemed less pronounced probably as a result of a research dip due to the COVID-19 pandemic). When looking at the use of the SCS, it is important to note that—notwithstanding our critical remarks—the majority of researchers (in 2020: 60% and in 2021: 70% as compared to 2003–2019: 71%) still employed the total score of this scale (which includes the reversed UCS or vulnerability items). In spite of the ever-increasing popularity of SCS and continued use of its total score, Neff (2022) recently re-opened the debate by publishing a commentary in which she made a new attempt to terminate the critique on her scale.

In her commentary, Neff (2022) tried to convince the scientific community that we (and others) do not properly understand the nature of bipolar continuums. To clarify her point, she made a comparison with temperature for which two qualitatively distinct opposites, warm and cold, are part of a prototypical bipolar continuum. This means that if the temperature shows a rise of one degree, this can be interpreted as an increase of warmth but at the same time can also be seen as a decrease of coldness, with both interpretations being valid as warm and cold are opposite qualities. In a similar vein, Neff (2022) argued that self-compassion should also be seen as “a bipolar continuum ranging from UCS (self-judgment, isolation, and over-identification) to CS (self-kindness, common humanity, and mindfulness), so that higher SCS [total] scores represent increased CS and reduced UCS” (p. 572).

However, the comparison between temperature and self-compassion is seriously flawed for at least two reasons. First and foremost, we are surprised (but also a bit amused) to hear Neff make the argument about the dimensionality of self-compassion as she already knows *from the very first beginning* that CS and UCS cannot be considered opposite qualities. In her original article in which she described the development and initial validation of the SCS (Neff, 2003b), she noted that a confirmatory factor analysis conducted on the three key elements of self-compassion—self-kindness versus self-judgment, common humanity versus isolation, and mindfulness versus over-identification—did *not* yield support for the expected one-factor model. On the contrary, she found evidence for two-factor models with separate CS and UCS components. In the discussion of this finding, Neff noted that “self-kindness and self-judgment are not mutually exclusive, so that having low levels of one behavior necessarily means having high levels of the other. A person may tend not to judge himself, but that doesn’t necessarily mean that he typically takes proactive steps to be kind to himself either. Likewise, an individual may rarely feel isolated in instances of failure, but that doesn’t necessarily mean she always puts her failure in the light of common human experience. In the same vein, just because one doesn’t tend to over-identify and run away with negative thoughts and emotions, it doesn’t necessarily mean that thoughts and emotions are held in mindful awareness (perhaps they are just ignored or repressed)” (Neff, 2003b, p. 234). We fully agree with this quote, which has also been supported by subsequent factor analytic research of the scale (see for a brief discussion Muris & Otgaar, 2019) as well as recent latent class studies revealing different response profiles to the SCS (e.g., Ulrich-French & Cox, 2020; Wu et al., 2021). However, of course, this runs fully counter to the bipolar continuum conceptualization of self-compassion that Neff (2022) has advanced in her latest commentary.

The second reason pertains to the fact that the assessment of a physical phenomenon such as temperature is totally different from the measurement of a psychological construct such as self-compassion. As we all know, temperature is caused by the kinetic energy of the particles (atoms or molecules) in a matter: the faster the particles move, the more energy they produce, and the higher the temperature of that matter will be. The other way around is the slower the particles of a matter move, the less energy they produce and the lower their temperature. Based on the fact that the decrease or increase of kinetic energy is also accompanied by subtle changes in the volume of a matter (i.e., increased motion is associated with increased volume and decreased motion with decreased volume), devices such as thermometers have been developed that enable us to measure temperature. The assessment of a psychological construct like self-compassion is far more complex and can certainly not be equated with measurements of, for example, temperature. The development of a scale essentially requires three basic steps: (1) formulation of a clear definition of the construct; (2) creating items that cover the emotional, behavioral, and cognitive components of the defined construct; and (3) testing the internal and external validity of the ultimate measure.

In case of the SCS, step 1—the formulation of a definition for self-compassion—was successful. Although there are alternative conceptualizations for self-compassion (see Strauss et al., 2016), Neff’s definition is plausible, easy-to-understand, and appealing to researchers because it covers a protective individual difference factor that fits nicely with contemporary views on human psychology that also focuses on positive concepts such as strengths, values, and resilience. In her first publication, Neff (2003a) noted that “Self-compassion involves being touched by and open to one’s own suffering, not avoiding or disconnecting from it, generating the desire to alleviate one’s suffering and to heal oneself with kindness. Self-compassion also involves offering nonjudgmental understanding to one’s pain, inadequacies and failures, so that one’s experience is seen as part of the larger human experience” (p. 87). From this multiplex definition, three key components were extracted, namely: “(a) self-kindness—being kind and understanding toward oneself in instances of pain or failure rather than being harshly self-critical, (b) common humanity—perceiving one’s experiences as part of the larger human experience rather than seeing them as separating or isolating, and (c) mindfulness—holding painful thoughts and feelings in balanced awareness rather than over-identifying with them” (Neff, 2003a, p. 85). Following this description, Neff explained the protective role of self-compassion as “an emotionally positive self-attitude that should protect against the negative consequences of self-judgment, isolation, and rumination (such as depression)” (p. 85). In this statement, she made a clear distinction between CS (protection) and UCS (vulnerability) and in its wake psychopathology.

During the construction of the questionnaire (step 2), Neff (2003b) somehow lost sight of the latter notion and fully embraced the idea of the dimensional nature of self-compassion by creating subscales for the three main components that consist of a mix of CS and UCS items. Although—as noted before—her initial study immediately revealed that CS and UCS items referring to the three key components did *not* constitute bipolar continuums, the fact that the compassionate and (reversed) uncompassionate components were substantially intercorrelated was sufficient for her to recommend the calculation of a total score representing *the overarching construct of self-compassion*.

However, in the past years, research has yielded substantial and convincing evidence showing that the inclusion of the UCS components seriously undermines the validity of the SCS (step 3). To begin with, studies have indicated that there are problems with the face validity of the scale as an index for measuring self-compassion as a protective psychological construct. Interviews about the content of the SCS have pointed out that CS is mainly indicating self-comforting and coping behavior, whereas UCS is predominantly reflecting emotional dysregulation, cognitive vulnerability, or even outright psychopathology (Muris et al., 2018; Zhao et al., 2021). Given this apparent split in the SCS, it is logical that we and other scholars (and even Neff herself, see Neff et al., 2018) began to explore the separate CS and UCS components to study their divergent relations to various psychological outcomes (e.g., Brenner et al., 2017, 2018; Coroui et al., 2018; López et al., 2015, 2018; Muris et al., 2018, 2019a), what has been named by Neff (2022) as the “differential effects fallacy in the study of self-compassion” (p. 572). However, the results of this research line have demonstrated that examining the effects of the separate CS and UCS components should not be dismissed as a scientific delusion but is essential to unravel the true protective nature of self-compassion and critical to examine the internal and external validity of the SCS (step 3).

In fact, it is the incorporation of UCS components in the SCS that seriously obstructs the investigation of the true effects of the vital shielding elements (i.e., self-kindness, common humanity, and mindfulness) of the self-compassion construct. To substantiate this point, Table 1 explains the contradiction between Neff’s (2003b, 2022) basic premise regarding the use of the SCS total score and our critical perspective on this procedure. As can be seen, we discuss the (unwanted) implications of Neff’s premise for various types of research designs that can be used to examine the role of self-compassion within the context of psychopathology and stress. We also provide a guideline of how these investigations can be improved by separating CS and UCS in the analysis of the data. To illustrate and further clarify our point, we now give some examples of studies that have successfully adopted this approach.

**Table 1:**
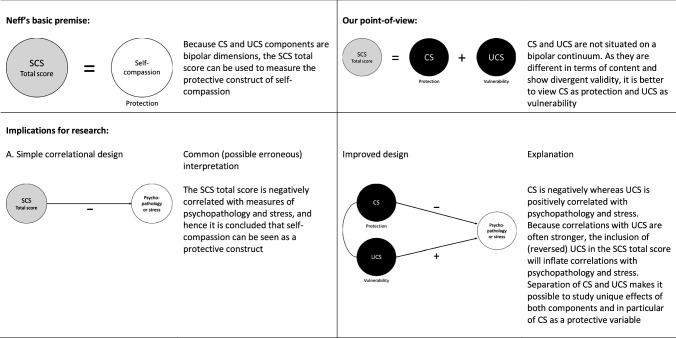

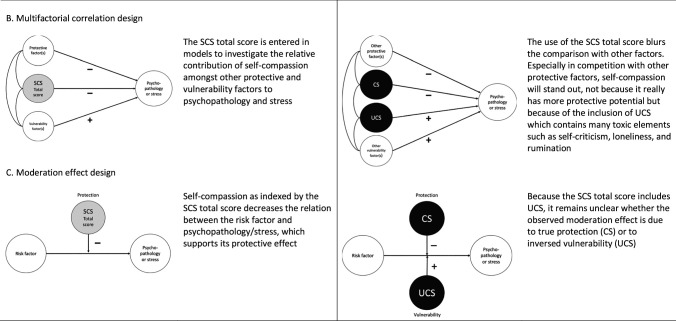

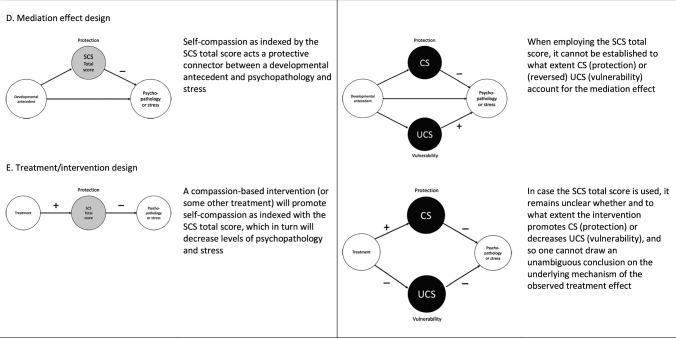
Neff’s basic premise regarding the use of the Self-Compassion Scale (SCS) as opposed to our perspective and a schematic overview of the implications of both perspectives for various types of research on the role of self-compassion in psychopathology and stress

*CS* compassionate self-responding, *UCS* uncompassionate self-responding

With regard to research relying on design A, there have been quite some studies demonstrating that the separation of CS and UCS reveals results that justify a somewhat different conclusion regarding the role of self-compassion in psychopathology and stress than when relying on the SCS total score. This was nicely shown in a study by López et al. (2018) who examined the relation between self-compassion as measured with the SCS and depressive symptoms in a large representative sample of community adults (*N* = 734) using a prospective correlation design. These researchers found substantial negative correlations between the SCS total score and depression on time 1 and time 2 (one year later), but also explored the predictive ability of the separate CS and UCS components on depressive symptoms both cross-sectionally and over the 1-year period. At the cross-sectional level, both CS and UCS were significantly associated with depressive symptoms, although UCS was by far the strongest correlate. At the one-year follow-up, only UCS emerged as a significant predictor of depressive symptoms. This made López et al. (2018) conclude that “the strong relationship between self-compassion and depressive symptoms [can] mainly be accounted for by the SCS negative items that measure a hard and cold response to the self, that is the exact opposite of self-compassion. This also implies that the positive experience of self-compassion, a kind and understanding response to the self, might only be weakly associated with depressive symptoms” (p. 1475).

Design B refers to the comparison of self-compassion with other vulnerability and protective factors in the prediction of psychopathology and stress. An example is our recent study (Muris et al., 2021) in which we examined the relative contributions of self-compassion—and its two components CS and UCS—and the basic personality traits of neuroticism and extraversion (study 1, *N* = 106) and self-esteem (study 2, *N* = 52) to symptoms of anxiety and depression in non-clinical adolescents. In both studies, it was found that the SCS total score consistently accounted for a significant proportion of the variance in anxiety and depressive symptoms. However, and of relevance, subsequent analyses in which we included CS and UCS as separate components revealed that it was mainly UCS that accounted for variance in these effects. The share of true self-compassion (CS) was fairly small and its contribution was even completely abolished when taking other relevant variables into account. On the basis of these findings, we concluded that researchers should decline from using the SCS total score to demonstrate the protective role of self-compassion within a context of psychopathology or stress, as “without proper investigation of the share of [CS] and [UCS], we simply do not know whether findings should be interpreted in terms of protection, vulnerability, or both” (Muris et al., 2021, p. 248).

A nice example of design C investigating the role of self-compassion as a moderator has been provided by the recent study of Li et al. (2022). This longitudinal study was conducted in a sample of 528 socioeconomically disadvantaged Chinese university students to explore whether self-compassion acted as a buffering variable in the association between perceived discrimination (a well-known life stressor) and psychological problems (as indexed by symptom measures of anxiety, depression, and stress). Moderation analyses were conducted with self-compassion as indexed by the SCS total score as well as with the separate CS and UCS scales. The results indicated that only CS consistently moderated the relationship between perceived discrimination and psychological problems, whereas when using the SCS total score or the UCS component, this moderating effect was not detected. Based on this result, the authors concluded that “when researchers investigate the moderation effect of self-compassion within the domains of psychopathology … or in the stressor-psychological distress link, CS may be better than overall [self-compassion] (total or average SCS score) in presenting a protective nature” (Li et al., 2022, p. 506).

Design D can be used when researchers want to investigate whether self-compassion acts as a mediating variable between a developmental antecedent on the one hand and psychopathology/stress on the other hand. For instance, based on the notion that attachment quality is a developmental factor involved in the etiology of psychopathology as well as in the formation of a self-soothing system, Brophy et al. (2020) examined the mediating role of CS and UCS in the relationships between two forms of attachment insecurity and depression as well as quality of life in a large population of German adults (*N* = 2253). Results were highly similar for depression and quality of life: attachment-related anxiety and avoidance both had a significant direct effect on these outcome variables. Most importantly, UCS consistently appeared to act as a mediator accounting for an indirect effect on depression and quality of life, whereas the indirect effect via CS either was of a negligible magnitude or did not attain statistical significance. These results showed that UCS is of greater importance than CS as a mechanism through which one’s attachment style affects depressive psychopathology and quality of life.

Self-compassion is also of special interest for clinicians because it would be amenable to therapeutic interventions. Psychological treatment in general (Mennin et al., 2013) and compassion-based interventions in specific (Ferrari et al., 2019) would foster compassionate self-responding which in turn would result in a decrease of psychopathology (see Table 1, Design E). In addition, treatment and intervention may also abolish uncompassionate self-responding and in this way result in a subsequent decline of psychopathology. Few studies have systematically evaluated the separate effects of interventions on CS and UCS and subsequent treatment outcome. One exception is a study by Eriksson et al. (2018) who examined the effects of a 6-week internet-based mindful self-compassion program in practicing psychologists (*N* = 101) who suffered from stress and burnout complaints. The participants were assigned to an intervention or a waiting list control condition, and before and after the treatment program, the SCS and measures of stress and burnout symptomatology were administered. The mindful self-compassion program was effective: statistically significant changes were noted for all measures: self-compassion as indexed by the SCS total score significantly increased while symptom scores significantly decreased in the intervention group, while no such changes could be noted in the waiting list condition. Further analyses revealed that the effect size of the change in CS was large, whereas that of the change in UCS was only moderate, which indicates that the treatment program was more effective in promoting compassionate self-responding than in reducing uncompassionate self-responding. Interestingly, however, when looking at the effects on treatment outcome (pre- to post-changes in stress and burnout symptoms), it appeared that changes in UCS were a better predictor than changes in CS. This suggests that interventions also need to focus on specifically reducing UCS as this may be crucial to the reduction of psychopathology and stress. A similar conclusion was reached by Wadsworth et al. (2018) in their study of 582 patients who were treated with intensive cognitive-behavior therapy or dialectical behavior therapy for their emotional problems. Here too, UCS was more substantially linked to treatment outcome, which made the authors conclude that “the negative aspects of self-compassion … may constitute an important target for treatment in acute settings” (p. 236).

With this reaction to Neff’s (2022) commentary, we have once again tried to explicate the shortcomings of using the SCS total score. As illustrated above, the employment of the total score will mask the true effects of self-compassion and hence muddy the waters of research on this potentially interesting protective variable (Neff, 2003a). However, Neff continues to be strongly attached to holding on to this total score. In her latest commentary, she even stated that “use of a total SCS score more comprehensively represents how taking a self-compassionate approach to suffering (i.e., increasing CS and reducing UCS) may affect outcomes such as life satisfaction, depression, and resilience” (p. 575). In our opinion, such blanket statements will not lead to a cumulative development of the research on self-compassion. Apart from the fact that empirical evidence consistently showed that separating UCS and CS can yield different results, focusing on and recommending researchers to only use the total score of the SCS is not in line with good psychometric practices. That is, such views lead to a lack of measurement transparency which is detrimental in examining the validity of a scale (Flake & Fried, 2020).

To conclude, we do not understand why Neff (2016a, 2016b, 2019, 2022) so tenaciously discards the notion of using the separate scores of CS and UCS in research as this would certainly give more insight in the distinct contributions of the protection and vulnerability components that are undeniably incorporated in her measure. Instead of defining our point of view as scientific nitpicking by some critical scholars who do not care for patients and other people with serious problems, she should embrace our idea rather than trying to promote her untenable ideas about the SCS total score and making false arguments about bipolar continuums and differential effects fallacies.
